# Photobiomodulation Associated With Conservative Treatment for Achilles Tendon Rupture: A Double-Blind, Superiority, Randomized Controlled Trial

**DOI:** 10.1016/j.arrct.2022.100219

**Published:** 2022-07-26

**Authors:** Pedro Rizzi de Oliveira, Lucas Simões Arrebola, Kelly Cristina Stéfani, Carlos Eduardo Pinfildi

**Affiliations:** a Department of Human Movement Sciences, Federal University of São Paulo (UNIFESP), Baixada Santista Campus, São Paulo; b Physical Therapy Department, Institute of Medical Assistance to the State Public Servant (IAMSPE), São Paulo; c Center of Technological Innovation, University of Sao Paulo Hospital of Clinics (HC-FMUSP), São Paulo, Brazil

**Keywords:** Achilles tendon, Low-level light therapy, Rehabilitation, ATR, Achilles tendon rupture, ATRS, Achilles Tendon Rupture Score, CI, confidence interval, ES, effect size, IAMSPE, Institute of Medical Assistance to the State Public Servant, NPRS, Numerical Pain Rating Scale, PBM, photobiomodulation, PBMG, photobiomodulation group, ROM, range of motion, SG, sham group, WALT, World Association for Photobiomodulation Therapy

## Abstract

**Objective:**

To investigate the effects of photobiomodulation on Achilles tendon rupture (ATR) treated conservatively.

**Design:**

Prospective, patient- and assessor-blinded, parallel, randomized controlled trial.

**Setting:**

Patients with acute ATR treated conservatively.

**Participants:**

Thirty-four male individuals with acute unilateral ATR treated conservatively (N=34), equally divided in 2 groups: photobiomodulation group (PBMG) and sham group, with mean age of 45.5±9.47 and 48.7±8.38 years, respectively.

**Intervention:**

All participants underwent through an immobilization period, followed by rehabilitation sessions (2 d/wk for 12 weeks) comprising strengthening, range of motion, and balance/weightbearing exercises. In PBMG, the tendon was irradiated with a photobiomodulation cluster (1 904 nm/50 mW infrared laser, 4 858 nm/50 mW infrared diodes, and 4 658 nm/40 mW red diodes; power density of 105 mW/cm^2^ per cluster area) during the immobilization period (2 d/wk for 8 weeks) and the sham group received a simulation of the procedure with no irradiation. Outcomes were assessed at the removal of the immobilization 12 and 16 weeks after tendon rupture.

**Main Outcome Measures:**

Primary outcome was the Achilles Tendon Rupture Score. Secondary outcomes included Numerical Pain Rating Scale at rest and during effort, plantar flexor strength, and ankle range of motion.

**Results:**

Both groups demonstrated an increase in the Achilles Tendon Rupture Score and improvements in range of motion, plantar flexor strength, and pain. There were no significant differences in outcomes between the 2 groups (*P*>.05) except in pain during walking, which was significantly lower in the PBMG in week 12 (*P*<.01, effect size=0.56) and week 16 (*P*<.01, effect size=0.55).

**Conclusion:**

Photobiomodulation associated with conservative treatment is not superior to conservative treatment alone for improving function in patients with acute ATR.

Achilles tendon rupture (ATR) is an orthopedic condition commonly related to sports.^1^ The main mechanisms of lesion are sudden ankle dorsiflexion, pushing off with the weight-bearing forefoot while extending the knee, and violent dorsiflexion of a plantarflexed foot (ie, landing from a jump).^2^

The incidence of this injury varies among countries, ranging from 6 to 37.3 per 100,000 inhabitants^3^ and is more frequent in men (3:1) with a mean age of 45 years.^4^

The management of this lesion can be either surgical or nonsurgical, both relying on initial immobilization in an equinus position of the ankle, gradually returning to a neutral ankle position.^5^ To date, there is no consensus on whether conservative or surgical approaches are superior, with both having similar functional outcomes in long-term follow-ups.^1^^,^^6^^,^^7^ Nonetheless, conservative treatment has lower complication rates^8^ and lower costs^9^ when compared to a surgical approach.

The immobilization period varies between 6 and 12 weeks and may lead to changes such as calf muscle atrophy, joint stiffness, longer time away from work, and late return to sports practice.^1^^,^^10^^,^^11^ Within this period, several important phases of the healing process take place and would represent window of opportunities for interventions aiming at repairing the tendon.

Photobiomodulation (PBM) is a tool often used to treat injured tendons/tendinopathy. The observed effects of PBM are believed to occur because of collagen synthesis and proliferation,^12^^,^^13^ neoformation of blood vessels,^14^ realignment of collagen fibers, and increased tensile force.^15^

There is evidence regarding PBM effects on tendon ruptures in rats^15^, ^16^, ^17^, ^18^; however, to date, there are no studies focused on verifying the effects of PBM in ATR in humans. Thus, this study aims to investigate the effects of PBM, delivered in the immobilization period, associated with a regimen of a rehabilitation program on ankle function in patients with ATR treated conservatively.

## Methods

### Study design

This was a double-blinded, superiority, parallel, randomized controlled clinical trial. This has been reported in accordance with the Consolidated Standards of Reporting Trials reporting statement.^19^

### Participants

Thirty-four male individuals (aged 18-65 years) with a clinical diagnosis of acute ATR were recruited from the Institute of Medical Assistance to the State Public Servant (IAMSPE). All participants signed an informed consent form. The study was approved by the Federal University of São Paulo Ethics and Research Committee on April 13, 2017 (reference: 2.012.302), and the IAMSPE Ethics and Research Committee on May 16, 2017 (reference: 2.065.907). It was prospectively registered in the Brazilian Clinical Trials Registry (RBR-845hhf).

This study was conducted in compliance with the principles of the Declaration of Helsinki.

### Eligibility criteria

#### Inclusion criteria

We included male individuals, aged 18 or older, with a clinical diagnosis of acute ATR requiring emergency care who were treated conservatively. Participants were required to exhibit a positive Thompson test (perceived when, after a manual calf squeeze, no movement is observed) along with the Simmonds triad (ankle in a dorsiflexed position when compared to uninjured leg, palpable gap, and a positive Thompson test).^20^

#### Exclusion criteria

We excluded people with bilateral tendon injuries and those with a history of prolonged anabolic/corticoid usage and previous tendon injury on the same leg as the ATR.

### Setting

This study was performed at a physiotherapy outpatient clinic at a tertiary hospital.

### Randomization

Concealed randomization was performed using a random numerical table generated at an online randomization service (www.randomization.com) by a physiotherapist who was not involved with the enrollment, assessments, or treatment. The randomized groups included the photobiomodulation group (PBMG) and the sham group (SG). Group allocation was conducted using opaque, closed, numbered envelopes. This groups allocation was then provided to the physiotherapist responsible for the intervention after the first assessment.^19^

### Intervention

SG participants were submitted to a cast immobilization protocol for 8 weeks (3 weeks in equine position, 3 weeks in a semineutral position, and 2 weeks in a neutral position, allowing weight-bearing). During that period, the participants were submitted to sham application of PBM twice a week. The cast was removed and a simulated application of PBM was performed. The procedure was performed with a blue opaque fabric placed over the application site. After each irradiation session, a new cast was manufactured.

**Fig 1:**
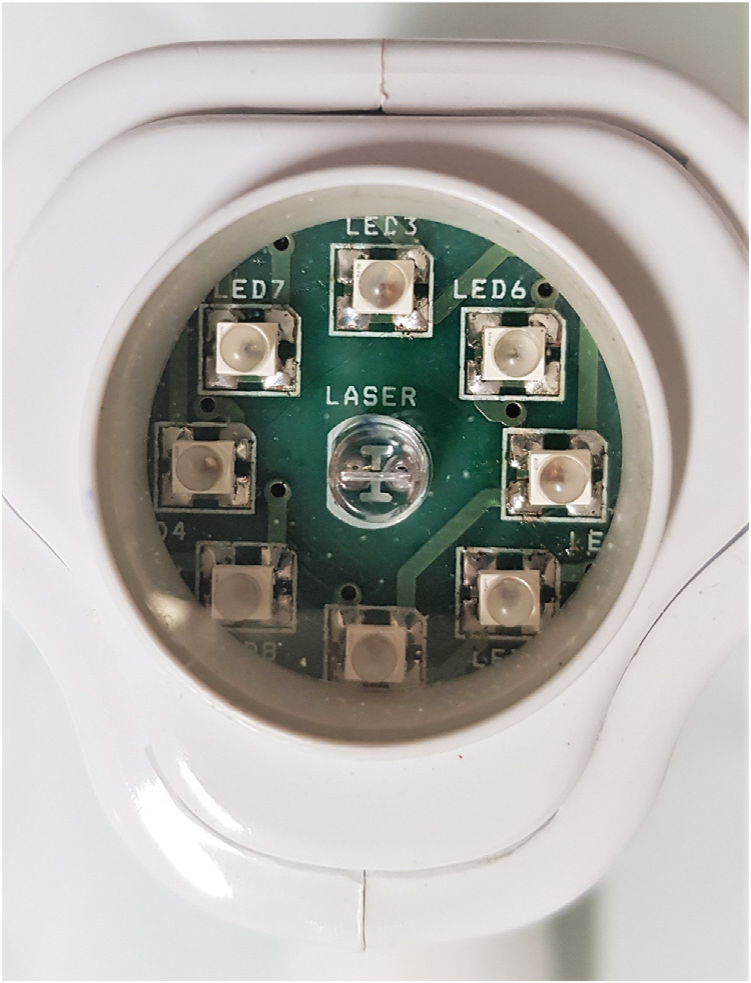
Photobiomodulation cluster.

**Fig 2:**
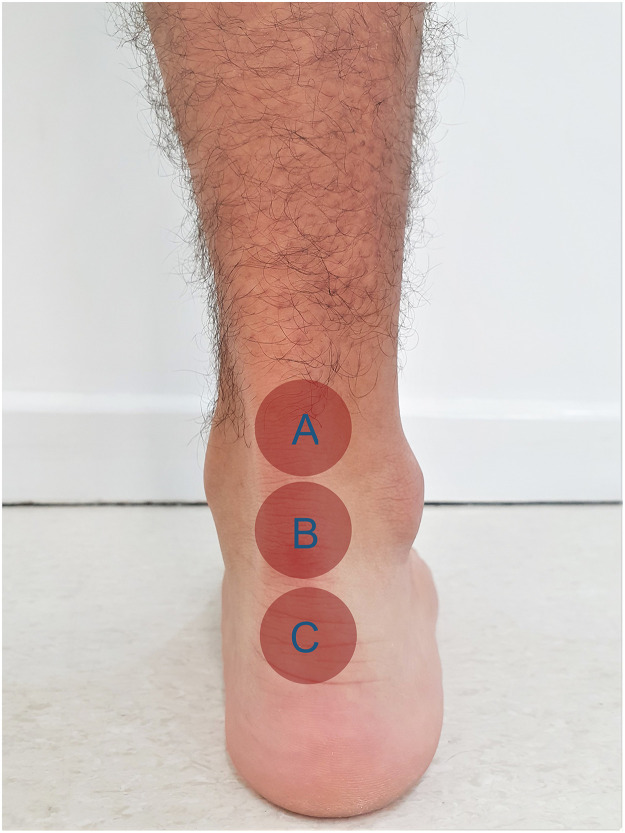
Photobiomodulation application sites. **(A)** Proximal region; **(B)** mid-portion region; **(C)** distal region, near calcaneus insertion.

PBM participants were submitted to the same immobilization protocol as the SG. During the immobilization period, the participants were submitted to PBM irradiation with a low-level PBM cluster (figure 1) twice a week, with the parameters described in table 1. The application site is shown in figure 2. The procedure was patient-blinded similar to the SG.

**Table 1:** Photobiomodulation cluster information

Wavelength (nm)	904 nm (AsGa)
	858 nm (AsGaAl)
	658 nm (AsGaInP)
Power (mW)	1 × 50 mW
	4 × 50 mW
	4 × 40 mW
Beam area (cm^2^)	904 nm (0.067 cm^2^)
	858 nm (0.130 cm^2^)
	658 nm (0.130 cm^2^)
Energy	
904 nm (AsGa)	5 J
858 nm (AsGaAl)	5 J
658 nm (AsGaInP)	4 J
Energy per session	168 J
Cluster area	5.72 cm^2^
Treatment time (per area)	1 min 30 s
Treatment time (total)	4 min 30 s
Power density of the cluster area (mW/cm^2^)	105 mW/cm^2^
Application technique	Stationary

At the end of the 8 weeks, both groups of participants had the cast definitively removed and began an 8-week rehabilitation protocol, ending the 16th week after treatment was initiated. The protocol consisted of strengthening and stretching exercises and is detailed in Appendix 1.

### Outcomes measures

**Fig 3:**
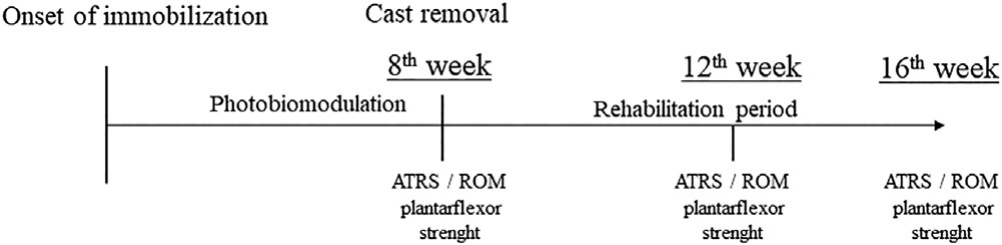
Treatment and assessment timeline.

Data were collected at the following time points: 8 weeks (immediately after immobilization was removed), 12 weeks, and 16 weeks after tendon rupture as illustrated in figure 3.

The primary outcome measure was the Achilles Tendon Rupture Score (ATRS), a questionnaire used to assess function in patients with ATR and validated in the Brazilian Portuguese language.^21^ The secondary outcomes were as follows:

- Numerical Pain Rating Scale (NPRS) at rest^22^ and during effort (walking).
- Ankle dorsiflexion and plantar flexion range of motion (ROM).
- Isometric plantar flexor muscles strength evaluation assessed using the Lafayette Manual Muscle Testing System Model 01165,^a^ factory calibrated, adapted from Sanada et al.^23^

The handheld dynamometer was placed inside a walking boot Robofoot^b^ and stabilized with an inelastic belt placed around the waist and the boot. The ankle was placed at a neutral position (90° plantar flexion). All patients were evaluated in sequence, alternating measurements between lower limbs to minimize fatigue. Before the evaluation, 2 submaximal contractions were performed to familiarize individuals. Subjects were verbally encouraged to perform the contractions at maximum capacity. For each leg, 3 measurements were performed with an interval of 30 seconds between tests. If the difference between measurements was greater than 10%, the result was discarded and remeasurement was performed. The muscle strength values obtained were normalized by body mass, employing the following formula: strength (kgf)/mass (kg) × 100. The mean of the 3 contractions was used for comparison.

All assessments were performed by the same physiotherapist who was blinded to the group allocation.

### Sample size calculation

The sample size was calculated for the primary outcome ATRS^21^ based on the results of Barford et al.^24^ The authors performed a clinical trial comparing 2 rehabilitation postoperative protocols (early mobilization vs immobilization) for ATR. Mean (SD) values for ATRS obtained 4 months after the injury were 57.1 (16.7) for early mobilization and 55.9 (17.0) for the immobilization protocol. Using these values as reference, for a power at 0.80 and with a statistical significance level of *P*<.05, a sample size of 32 individuals was needed. To accommodate for expected dropouts, 17 per group was calculated, and a total of 34 participants were recruited.

### Statistical analysis

Statistical analysis was performed using RStudio v1.4.1717.^c^ Descriptive statistics of demographic data are represented as mean±SD and frequency. Assumption of data normality was assessed by the Shapiro-Wilk test.

Analysis of the group differences of the primary outcome ATRS was done by a linear mixed-effects model. The considered fixed effects were group, time, and group × time interaction. Patient number identification was used as a random effect. The initial model considered varying intercept and slope but provided a poor fit. Therefore, a second model with varying intercept and fixed slope was considered.

The analysis of the differences between groups at 8 (immediately after the immobilization was removed), 12, and 16 weeks were performed by independent sample *t* test (for normally distributed data) and Mann-Whitney test (for nonnormally distributed data). Effect size rank biserial correlation was used for the effect size (ES), with associations considered weak below 0.10, moderate between 0.10 and 0.49, and strong between 0.50 and 1.00. All statistical tests considered an alpha set to .05.^25^

The analysis followed an intention-to-treat principle. Missing data were addressed through multiple imputation and analyzed through sensitivity analysis.^26^ The imputations were analyzed through descriptive statistics (mean±SD, interquartile ranges, and confidence intervals) to verify that the imputed data did not deviate significantly from the original.

## Results

Forty patients were assessed for eligibility from June 2017 to March 2020. Thirty-four were randomized: 17 were allocated to PBMG and 17 to SG. One patient from each group was lost to follow-up. Demographic data for both groups are shown in table 2.

**Table 2:** Demographic data

	PBMG	SG	*P* Value
Patients, n (%)	17 (50)	17 (50)	
Age (y)			
Mean±SD	45.5±9.47	48.7±8.38	.30
Height (cm)			
Mean±SD	174±6.12	174±10.0	.87
Weight (kg)			
Mean±SD	83.8±11.1	84.0±15.7	.96
Body mass index			
Mean±SD	27.7±3.2	28.0±3.0	.80
Injured side, n (%)			
Right	7 (41)	8 (47)	
Left	10 (59)	9 (53)	

### Primary outcome

#### ATRS

**Fig 4:**
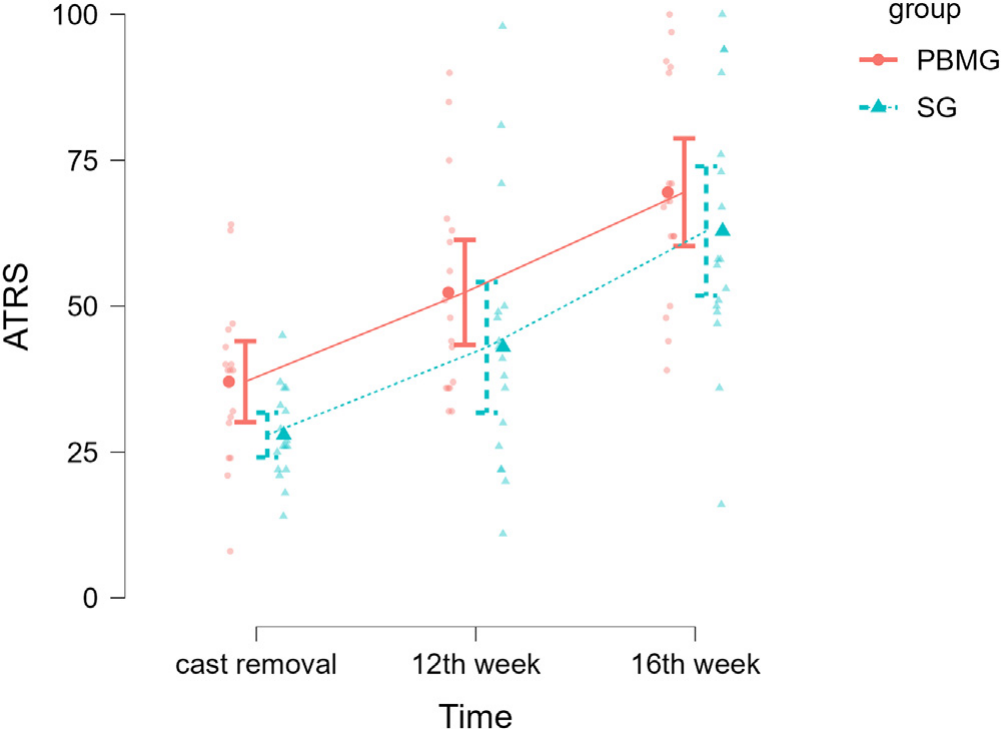
Comparison between groups of ATRS at times represented by mean and standard error.

ATRS scores over time are shown in figure 4. We fitted a linear mixed model to predict ATRS with group and time. The model's total explanatory power was substantial (conditional *R*^2^=0.67) and the part related to the fixed effects alone (marginal *R*^2^) was 0.40. Within this model, the effect of group was statistically nonsignificant (*P*=.176) and the effect of time was statistically significant (*P*<.01) as shown on table 3. No interaction between factors was observed.

**Table 3:** Primary outcome analysis of the ATRS group difference between PBMG and SG according to the mixed-effect model

Predictors	Estimates	CI	*t* Value	*P* Value
Intercept	31.37	6.33-56.41	2.49	<.01*
Group	−10.86	−26.7 to 4.97	−1.36	.176
Time	15	5.03-24.97	2.99	<.01*
Group*Time	1.24	−5.07 to 7.54	0.389	.698

⁎ *p* <.05.

### Secondary outcomes

Results for the secondary outcomes are summarized in table 4.

**Table 4:** Secondary outcomes, mean and SD

	PBMG	SG	*P* Value	Effect Size [95% CI], rank correlation
8 weeks				
NPRS at rest	0.41±1.28	1.82±2.60	.051	0.308 [−0.075 to 0.612]
NPRS during walking	3.54±3.57	5.06±3.09	.313	0.204 [−0.185 to 0.538]
Ankle dorsiflexion–difference between legs (°)	12.7±6.28	14.32±811	.388	0.176 [−0.212 to 0.517]
Ankle plantar flexion–difference between legs (°)	8.82±6.90	8.88±6.07	.89	0.031 [−0.348 to 0.401]
Isometric plantar flexor muscle strength–difference between legs (kgf/kg*100)	43.77±20.32	45.73±18.90	.772	0.100 [−0.573 to 0.772]
12 weeks				
NPRS at rest	0.88±1.41	1.24±1.71	.607	0.097 [−0.288 to 0.455]
NPRS during walking	2.79±2.01	5.29±2.73	.005*	0.564 [0.240-0.775]
Ankle dorsiflexion–difference between legs (°)	6.53±3.9	6.65±4.73	.959	0.014 [−0.363 to 0.386]
Ankle plantar flexion–difference between legs (°)	9.88±6.84	9.58±5.84	.917	0.024 [−0.354 to 0.395]
Isometric plantar flexor muscle strength–difference between legs (kgf/kg*100)	33.25±15.70	27.02±13.28	.221	−0.428 [−1.105 to 0.256]
16 weeks				
NPRS at rest	0.29±0.68	0.79±0.95	.075	0.298 [−0.087 to 0.605]
NPRS during walking	1.71±1.90	3.91±2.20	.006*	0.554 [0.226-0.769]
Ankle dorsiflexion–difference between legs (°)	4.12±2.34	4.06±2.54	.807	−0.052 [−0.418 to 0.329]
Ankle plantar flexion–difference between legs (°)	8.65±5.81	7.76 ± 7.17	.425	−0.163 [−0.506 to 0.226]
Isometric plantar flexor muscle strength–difference between legs (kgf/kg*100)	32.41±13.71	28.67±13.66	.431	−0.273 [−0.947 to 0.404]

There was no substantial difference between the SG and PBMG for the NPRS at rest at any time point. For NPRS during effort, significant differences were found at week 12 (ES=0.564; 95% confidence interval [CI], 0.240-0.775; *P*=.005) and 16 (ES=0.554; 95% CI, 0.226-0.769; *P*=.006). As for ankle ROM and plantar flexor muscle strength, no significant difference was found at any time point.

## Discussion

This is the first double-blind randomized controlled trial to investigate the effects of PBM on ATR in humans. The main findings of the study were that (1) patients with ATR treated conservatively undergoing PBM showed no differences in function, as measured by the ATRS, when compared with individuals undergoing simulation; (2) patients with ATR undergoing PBM have less pain during walking when compared with those undergoing PBM simulation; (3) there was no influence of PBM on muscle strength; and (4) individuals with ATR submitted to PBM did not have different ankle ROM than those submitted to sham.

The use of PBM immediately after tendon rupture was the foundation of this translational study because the literature shows that the initial phase of the injury (acute and subacute phase) is essential to allow an effect of treatment aimed at improving repair. However, these studies are experimental and were developed in animal models with partial or total Achilles tendon injury. de Jesus et al^27^ carried out an experiment in rats to evaluate the effects of PBM (780 nm) using 3 different time treatment durations (PBM delivered in 1 day, for 3 days, and 7 days in a row) on the repair of injured tendons in these animals. The authors found a greater amount of collagen type I and regular fiber alignment in the group that underwent PBM when compared to a control group. Carrinho et al^28^ also observed similar effects with a greater amount of type I collagen and better tendon tissue realignment in animals treated with PBM (830 nm and 685 nm).

Function measured by ATRS was not influenced by PBM. ATRS values at week 12 are in agreement with those obtained in the study by Olsson et al,^29^ who compared surgical treatment with an accelerated rehabilitation protocol (43±20) vs conservative treatment (35±14). There are no studies measuring ATRS at the 16th week, but there is a tendency for values to level off in late assessments.^29^, ^30^, ^31^

PBM had a significant effect on NPRS measured during walking from the 12th week (EF=0.564; 95% CI, 0.240-0.775; *P*>.01) to the 16th week (ES=0.554; 95% CI, 0.226-0.769; *P*>.01). Though pain was not the primary outcome of the present study and the sample size calculation was made considering ATRS, a post hoc analysis was performed and verified an achieved power of 0.82 at week 12.

The analgesic effects of PBM in humans have been the subject of 2 systematic analyses, both relating the efficacy of the therapy to the dose chosen. Stausholm et al^32^ verified that in knee osteoarthritis, PBM doses between 4 and 8 J with a wavelength of 785-860 nm reduced pain and disability. Clijsen et al^33^ performed a systematic analysis investigating the effects of PBM on pain in several musculoskeletal disorders and observed that studies that utilized dosage recommended by the World Association for Photobiomodulation Therapy (WALT)^34^ yielded better pain relief compared to those that did not follow WALT's recommendation.

The PBM dose used in this study is in agreement with that recommended by WALT for tendinopathy because there is no recommendation for tendon rupture treatment. In a systematic review conducted by Tumilty et al,^35^ energy density values varied substantially from 1.4 to 150 J/cm^2^ for tendinopathy treatment. Therefore, the dose setting of this study is in accordance with the literature and WALT.

For the PBM treatment, we used infrared (858 nm) and red (658 nm) light emitting diodes as well as an infrared (904 nm) laser. This allowed us to cover the light wavelengths used in experimental studies^17^^,^^28^ and in clinical trials.^35^^,^^36^ We hypothesized that irradiation of a ruptured tendon with the wavelengths mentioned above could lead to an increase of collagen type I, fiber realignment, and increase in tendon vascularization, therefore indirectly affecting function and performance because those effects are known to occur in animals submitted to PBM.^17^^,^^27^^,^^37^ However, that was not the case in the actual study because we found no difference between groups regarding function, ROM, and strength.

### Limitations

The study has some limitations. Firstly, we were not able to measure or verify structural changes in tendons through imaging such as magnetic resonance imaging because of limitations in the hospital where the study was conducted. Secondly, only the patient and the assessing therapist were blinded. We were not able to blind the therapist who delivered PBM therapy because the device we used did not have that function. Finally, we performed sample size calculation based on a previous study^24^ that had a similar design regarding ATR treated conservatively and that used ATRS as a primary outcome because, to date, there is no information about the ATRS minimally clinically important difference and SD for ATR.

## Conclusions

Despite a statistically significant influence on pain during walking, PBM has no effect on function in patients with ATR treated conservatively. Future studies may determine the role of PBM in tendon structure and morphology in patients with ATR.

## Suppliers

a. Lafayette Manual Muscle Testing System Model 01165, Lafayette Instrument Co.

b. Robofoot, Salvapé Produtos Ortopédicos LTDA.

c. RStudio v1.4.1717, PBC.

## Appendix Supplementary materials

Image, application 1

## Footnotes

Supplementary material associated with this article can be found, in the online version, at doi:10.1016/j.arrct.2022.100219.
