# Supplementary material for: Photobiomodulation Associated With Conservative Treatment for Achilles Tendon Rupture: A Double-Blind, Superiority, Randomized Controlled Trial
Source: Arch Rehabil Res Clin Transl. 2022 Jul 26;4(4):100219. doi: 10.1016/j.arrct.2022.100219 (PMC9761261; doi:10.1016/j.arrct.2022.100219)
Supplement: Supplementary file 1 [file mmc1.docx]

Appendix 1

|  | Immobilization Period | 9th-10th Week | | 11th-12th Week | | 13th-14th Week | | 15th-16th Week | |
| --- | --- | --- | --- | --- | --- | --- | --- | --- | --- |
| Strength exercises |  | Bilateral calf raise | 3 Sets 15 repetitions | Bilateral calf raise | 3 Sets 15 repetitions | Bilateral calf raise | 3 Sets 15 repetitions | Bilateral calf raise | 3 Sets 15 repetitions |
|  |  | Bilateral calf raise on leg-press 45° | 3 Sets 15 repetitions | Bilateral calf raise on leg-press 45° | 3 Sets 15 repetitions | Unilateral calf raise on leg-press 45° | 3 Sets 15 repetitions | Unilateral calf raise on leg-press 45° | 3 Sets 15 repetitions |
|  |  | Sitting 1-leg calf exercise with elastic band | 3 Sets 20 repetitions | Sitting 1-leg calf exercise with elastic band | 3 Sets 20 repetitions | Sitting calf raise | 3 Sets 15 repetitions (no added weight) | Sitting calf raise | 3 Sets 15 repetitions (no added weight) |
| ROM exercises |  | Closed kinetic chain ROM training | 10 Sets 20 s | Closed kinetic chain ROM training | 10 Sets 20 s | Closed kinetic chain ROM training | 10 Sets 20 s | Closed kinetic chain ROM training | 10 Sets 20 s |
| Balance exercises |  | Gait training on treadmill | 5 Min/3 km/h | Gait training on treadmill | 5 Min/3 km/h | Gait training on treadmill | 5 Min/4 km/h | Gait training on treadmill | 5 Min/4 km/h |
|  |  | Unilateral stance on regular surface | 5 Sets 60 s | Unilateral stance on regular surface | 5 Sets 60 s | Unilateral stance on proprioceptive disk | 5 Sets 60 s | Unilateral stance on proprioceptive disk | 5 Sets 60 s |
